# Factors associated with obesity and metabolic syndrome in ageing black South African women

**DOI:** 10.1080/16549716.2017.1359922

**Published:** 2017-10-10

**Authors:** Philippe Jean-Luc Gradidge

**Affiliations:** ^a^ Centre for Exercise Science and Sports Medicine (CESSM), Faculty of Health Sciences, University of the Witwatersrand, Johannesburg, South Africa

**Keywords:** Obesity, metabolic syndrome, South Africa, women, behavioural determinants, body-size perception, adiponectin

## Abstract

**Background**: The incidence of obesity and related metabolic diseases is high and increasing in sub-Saharan African women. Evidence on the determinants of these diseases is limited, particularly in black South African women.

**Objective**: This PhD review attempts to understand the determinants of obesity and metabolic syndrome (MetS) in a population of ageing urban-dwelling black South African women.

**Methods**: Drawing on the longitudinal Birth-to-Twenty-Plus cohort, data were collected in 2002/03 and 2012/13, including information on behavioural factors (smoking, sitting time, physical activity, smokeless tobacco, and alcohol consumption), body-size perception, body composition (measures of adiposity and lean mass), blood pressure, and cardiometabolic biomarkers (lipid profile, fasting insulin, fasting blood glucose, insulin resistance, leptin, and adiponectin).

**Results**: The prevalence of obesity and related cardiovascular disease risk was high and increased significantly over the 10 year period. Despite most of the study population being physically active, sitting time was high and associated with elevated blood pressure and hypertriglyceridaemia. Two groups of people were observed, those who were happy and those who were unhappy with their body size. In logistic regression analysis, the risk of MetS was lowered by abdominal subcutaneous adipose tissue and adiponectin, and increased by age, smoking, truncal lean mass, and insulin resistance.

**Conclusions**: Obesity was confirmed to be increasing in black South African women, despite most women being sufficiently active according to guidelines of ≥150 min activity/week. Nevertheless, the contribution of sitting time to poor health outcomes is evident in this study population and must be addressed, particularly in women who are content with being obese. The novel finding of the effects of abdominal subcutaneous tissue and truncal lean mass with MetS requires further investigation. The protective effect of adiponectin against MetS is an important finding which highlights the novel interaction between adiposity and cardiometabolic diseases in black South African women.

## Background

Obesity is known to be increasing in sub-Saharan Africa [1]. This phenomenon has resulted in a dramatic increase in the risks associated with obesity-related cardiovascular diseases in South Africans [2]. This is not unique to South Africa, but is also characteristic of other countries on the African continent and many developing nations around the world [3,4]. However, within the sub-Saharan African region, obesity is most pronounced in black South African women, and South Africa is still experiencing a rapid rural–urban shift which has promoted cardiometabolic diseases related to fat accumulation [5]. The global movement of populations from rural to urban locations is driven largely by economic reasons; however, in South Africa people living in the urban setting are at greater risk of obesity and related disorders, and this is more noticeable in black South African women than in men and their white counterparts [6].

This PhD review describes selected factors associated with the increase in obesity in South African women. Black South African women are supposed to have better access to health; however, they are still generally poorer and less educated, and have limited resources compared with the other ethnic groups in the country [2]. Rural-dwelling black South African women also have better activity profiles than urban-dwelling black women, resulting in lower disease risk [7]. With the continual shift of populations to the urban setting, beliefs around acceptable body size are being challenged; such that some obese black South African women perceive their body size to be too large. In the rural setting, being overweight is still associated with wealth and prosperity [7]. Even though these behavioural and psychosocial factors may pose potential challenges for lifestyle modification, they are not yet fully understood. Evidence on the aetiology of obesity and metabolic syndrome (MetS) in African populations is very limited [8]. The black South African female population can provide researchers with some insight, as the prevalence of MetS in this cohort was observed to be high [9]. A study of MetS is also relevant given the Global Burden of Disease initiative, which has highlighted some key cardiometabolic diseases and behavioural risk factors as global medical targets. Therefore, the purpose of this paper is to improve our understanding of the interrelationship of the determinants of obesity and MetS in black South African women. The results presented here originate from cross-sectional and longitudinal studies linked to the doctoral research, and are organised by theme: (1) patterns and correlates of self-reported physical activity; (2) the role of selected lifestyle behavioural and body-size perception factors in determining changes in anthropometry; and (3) metabolic and anthropometric risk factors associated with MetS [10–12].

### Conceptual framework

**Figure 1.**
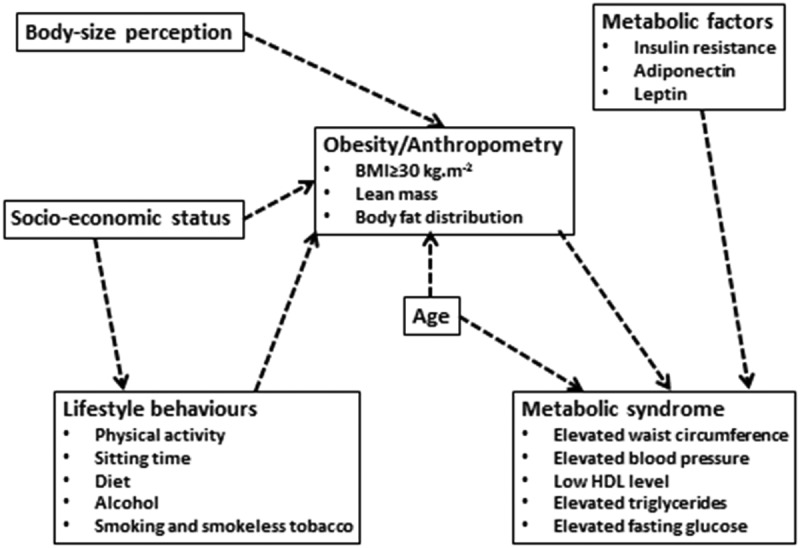
A conceptual, hypothetical framework of the proposed links between behaviours, body composition, and metabolic syndrome [13]. HDL, high-density lipoprotein cholesterol.

The conceptual framework shown in Figure 1 suggests that obesity is an essential risk factor in the development of cardiometabolic diseases, indicating the need to determine the underlying risk factors involved [13]. There are very few data on the physical activity behaviour of African populations [14], and the influence of body-size perception on obesity has not been confirmed in adult African women [7]. Dietary patterns have an influence on the role of fat accumulation. For the purpose of this paper, the influence of high-fat food consumption was investigated, particularly as the consumption of foods high in fat is associated with an increased risk of obesity in black South African women [15]. Evidence shows a positive association between obesity and body-size perception in adolescent African girls, and the same association between physical inactivity and body mass index (BMI). Sitting time is well known to have a positive role in adiposity [16,17], and is a good proxy measure of sedentary activities. Socio-economic status (SES) is known to have a positive association with obesity; however, its association with physical activity in African women is unknown. The aetiology of MetS in African populations is also not clear. Therefore, the framework also depicts the possible influence of SES, lifestyle behaviours, body-size perception, and metabolic biomarkers on MetS. Smokeless tobacco and alcohol consumption have been shown to have a role in obesity and cardiometabolic diseases [18,19]. With increasing fat accumulation, the level of adiponectin (a hormone secreted from adipose tissue) is reduced, suggesting that it may have a role in MetS [20]. Identifying the risk factors influencing body composition and MetS is fundamental to understanding the pathogenesis of these diseases in this group of African women, who seem to be more susceptible to this phenomenon than white women [7].

## Methods

### Study setting and population

The Soweto metropolitan region is 15 km south-west of central Johannesburg. The area was initially created as a separate district to accommodate the black African miners who worked in the surrounding gold mines [22]. The region is now mostly populated by black South Africans, most of whom are female, originate from the isiZulu ethnic group, and speak a native home language as well as conversational English [23]. Approximately 40% of Johannesburg’s total population reside in Soweto, and the 2011 census estimates the population size at around 1.27 million. The total area of Soweto is approximately 200 km^2^ and includes 34 sub-districts, with most houses having being built with bricks [22].

**Figure 2.**
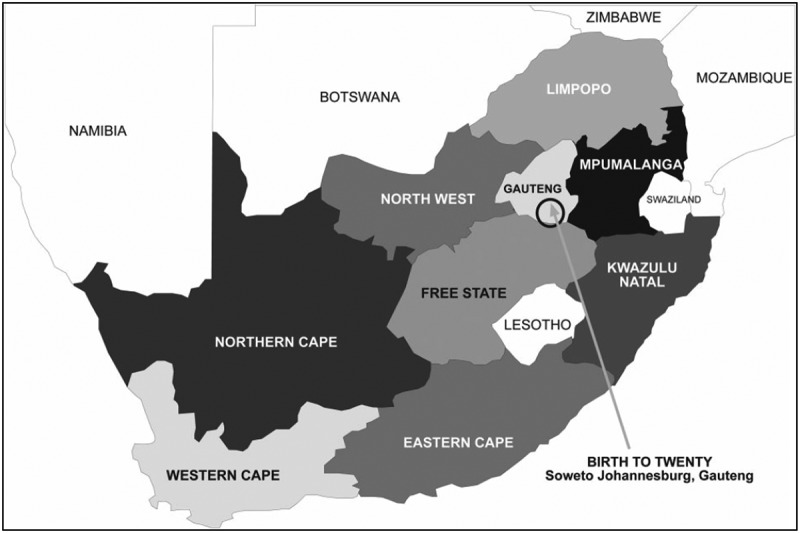
Physical activity patterns by sedentary behaviour-promoting household assets. MVPA, moderate–vigorous physical activity; TV, television. Source of data: Gradidge et al. (2014) [10]. **p < 0.005, ***p < 0.0005 vs women who do not own motor vehicles or TVs.

The Birth-to-Twenty-Plus (Bt20) cohort study, with a total sample of over 3000 participants, commenced in 1990 in the Soweto, Johannesburg region [24]. Pregnant women were recruited and asked about their health and social circumstances [9]. An attrition rate of 30% occurred over 20 years, predominantly following the health and social development of the biological mothers/caregivers and their children [25]. The Bt20 sample is representative of families that have remained residents of Soweto for over 20 years, with approximately equivalent representation of males and females. In total, 2200 caregivers/mothers remain in contact with the administrators of the study, resulting in the Bt20 study being the leading longitudinal study of African health and development on the continent [25]. The data were collected from this study cohort at facilities located in Soweto, Johannesburg. The baseline data for this paper were collected in 2002/03 (*n* = 977) and the follow-up data were collected 10 years later (*n* = 702). About 43.8% of the respondents had data from the two data-collection waves.

### Explanatory variables

#### Physical activity and sitting time

The data were collected at both time-points. Domains of physical activity (work, walking for transport, and recreation-related spheres of activity) were determined by the Global Physical Activity Questionnaire (GPAQ) [10,26]. Sitting time, a proxy measure of sedentary behaviour, was determined by the same questionnaire. Using GPAQ data from the three main domains of physical activity, participants were stratified into a GPAQ physically active group if the following criteria were met: a minimum of 150 min on at least 5 days of the week, or a minimum of 60 min of vigorous physical activity on at least 3 days of the week; or a minimum of 600 metabolic equivalent minutes on a least 5 days of the week from combined moderate and vigorous physical activity (MVPA) [10].

#### Body-size perception

Data on body-size discrepancy and dissatisfaction were collected at both time-points and were used as proxy of psycho-social measures. Nine silhouettes, ranging from ‘underweight’ (1 = silhouettes 1 and 2) to ‘normal weight’ (2 = silhouettes 3–5), ‘overweight’ (3 = silhouettes 6 and 7), and ‘obese’ (4 = silhouettes 8 and 9), were used to determine perception of body size [11]. Participants chose two silhouettes: one which best matched their ‘current’ weight status, and one which corresponded to their ‘ideal’ weight status. The nine silhouettes were used in both instances, i.e. for body-size discrepancy and for body-size dissatisfaction, and thoroughly shuffled before every participant interview. Body-size discrepancy was calculated by ‘perceived’ minus ‘actual’ BMI–weight discrepancy score. The BMI–weight categories were recoded as 1, 2, 3, and 4 to represent underweight, normal, overweight, and obese categories, respectively. The body-size discrepancy scores were categorised into (a) underestimation (negative scores), (b) overestimation (positive scores), and (c) happiness (zero score) with body size. Body-size dissatisfaction of participants was determined by subtracting the participants’ current perception of their body shape from how they wanted to look. A longing to be thinner is represented by positive scores, zero scores denote satisfaction with body size, and a wish for larger body size is indicated by negative scores.

#### Dietary intake, alcohol consumption, and smoking status

A questionnaire was used to determine consumption of high-fat foods (‘fatty cuts of red meat, chicken with skin, full-cream milk, processed meats, crisps, and fried food items’), alcohol intake, and smoking status (including snuff/smokeless tobacco consumption) [11,12]. Data on high-fat foods and alcohol consumption were collected at baseline in 2002/03, while smoking was collected at both time-points. Snuff consumption was only recorded at the second time-point, in 2012/13.

#### Biochemical analyses

Fasting blood samples were collected and analysed for fasting glucose, fasting insulin, insulin resistance [measured by homeostasis model assessment (HOMA)], serum triglycerides, high-density lipoprotein (HDL) cholesterol, low-density lipoprotein (LDL) cholesterol, total cholesterol, adiponectin, and leptin [10,12]. Biochemical data were collected at both time-points.

### Outcome measures

BMI and waist circumference were obtained using standardised methods, and universal definitions were used to define BMI (≥ 30 kg/m^2^) and waist circumference (≥ 80 cm) as obesity [27]. Dual-energy X-ray absorptiometry (DXA) was used as a more robust method of body composition [10,11]. Anatomical sites were used as markers in DXA to determine cut-off lines for central and peripheral fat [28]. In addition, the changes in these body-composition variables were adjusted by including the matching baseline body-composition variable in the longitudinal study. All body-composition data were collected at both time-points, except for visceral adipose tissue and subcutaneous adipose tissue, which were determined by ultrasonography at the second time-point. Cardiometabolic components of MetS were defined using the harmonised guidelines, i.e. systolic blood pressure ≥ 130 mmHg, diastolic blood pressure ≥ 85 mmHg, fasting blood glucose ≥ 5.6 mmol/l, triglycerides ≥ 1.7 mmol/l, HDL cholesterol < 1.3 mmol/l, LDL cholesterol ≥ 3 mmol/l, and total cholesterol ≥ 5 mmol/l [29]. MetS was defined as three or more out of four components, without waist circumference, to uncover the association of anthropometric compartments with the syndrome and its components [12].

### Confounding variables

Household SES index was collected at baseline only and determined using a standardised questionnaire, which ranked 12 household commodities according to the cost of the item. Thus, households were ranked from highest to lowest cost as owning (1) motor vehicle, (2) satellite television (TV), (3) washing machine, (4) mobile phone, (5) microwave, (6) refrigerator, (7) telephone, (8) Electron Media Network, (9) second TV, (10) electricity, (11) video, and (12) radio, to produce an overall SES score which ranged from ownership of no household items (or an SES score of 0) to ownership of all household items (or an SES score of 78) [30]. Total SES score was used as a continuous variable in multivariable linear regression models for subtotal fat and lean mass. In addition, TVs and motor vehicles were seen as household assets which encourage sedentary behaviour [31,32], and were analysed as separate constructs in further analyses [10]. Level of education was stratified as ‘no education’, ‘completed primary school but did not attend high school’, ‘attended high school but did not graduate’, and ‘completed high school’, and collected at both time-points [11]. Age was used as a continuous variable at both time-points.

### Analyses

#### Descriptive analysis

Patterns of physical activity were described in active and inactive women, and by household assets which were noted as sedentary behaviour-promoting assets (ownership of motor vehicles and/or TV). Body composition was described by activity group, body-size perception factors, and change in body composition (calculated as absolute change from baseline to 10 year follow-up) [10,11].

#### Multivariate linear regression analyses

Regression models were conducted to determine the contributors of body composition and cardiometabolic diseases. All models for the cardiometabolic outcomes initially included age, SES, subtotal fat mass, lean mass, waist circumference, sitting time, total MVPA, work MVPA, leisure MVPA, and walking for travel. The change models for anthropometry were adjusted for age, SES, dietary intake, alcohol consumption, smoking, and education, while the baseline anthropometry models were adjusted for age, SES, fat mass (for the waist circumference model), sitting time, total MVPA, work MVPA, leisure MVPA, and walking for travel. Only those explanatory variables with a *p*-value < 0.1 were included in the regression models. Adjusted *β* coefficients are reported in Table 1 [10,11].

**Table 1.** Multivariable linear regression models for baseline and change in anthropometry outcomes.

Covariate	Regression coefficients (*β*) for outcomes								
	BMI^a^	WC^b^	WC^a^	Subtotal fat^b^	Subtotal fat^a^	Lean mass^b^	Lean mass^a^	Central fat^a^	Peripheral fat^a^
Age	–	0.21***	–	0.15***	–	0.002	−0.14*	–	–
Alcohol consumption (≥3 drinks/day)	–	–	−0.15**	–	–	–	–	–	–
Household SES score	–	–	–	0.08**	–	0.05	–	–	–
Active smoker	–		–	–	–	–	−0.14*	–	–
Participants who underestimated actual body size	–	–	–	–	−0.16*	–	–	–	−0.15*
Work MVPA (min/week)	–	–	–	–	–	0.06*	–	–	–
Vigorous PA (min/week)	−0.11*	–	−0.15**	–	−0.12*	–	–	−0.15*	−0.13*

^a ^Change in body composition, adjusted for matching baseline body composition variable [age, socio-economic status (SES), dietary intake, alcohol consumption, smoking, and education]; ^b^ baseline body composition, adjusted for age, SES, fat mass [for the waist circumference (WC) model], sitting time, total moderate–vigorous physical activity (MVPA), work MVPA, leisure MVPA, and walking for travel. Only those independent variables significant in the bivariate analysis were included in the final models shown in this table.

–, Not included in the model; BMI, body mass index; PA, physical activity.

*p < 0.05; **p < 0.005; ***p < 0.0005.

Source of data: Gradidge et al. (2014, 2015) [10, 11].

#### Logistic regression analyses

Logistic regression models were used to determine the factors associated with MetS and its components. The following scientifically plausible variables were included in the initial bivariate logistic regression analysis: HOMA, adiponectin, trunk fat-free soft-tissue mass (FFSTM), subcutaneous abdominal fat thickness, waist and hip circumference, total body fat mass, total body FFSTM, subcutaneous and visceral adipose fat thickness, menopausal status, receiving anti-retroviral medication, and smoking. Backward, stepwise removal of non-significant variables (*p* > 0.05) was used to determine which of these variables remained as independent variables in the logistic regression models. Significant odds ratios with accompanying 95% confidence intervals are reported in Table 2 [12].

**Table 2.** Multivariable linear regression models for baseline metabolic variables.

Covariate	Regression coefficients (*β*) for outcomes							
	Fasting glucose	Fasting insulin	HDL	LDL	Total cholesterol	Triglycerides	SBP	DBP
Age	0.10**	−0.07*	−0.05	0.28***	0.25***	0.08*	0.24***	0.13***
Sitting time (min/week)	–	–	–	–	–	0.12***	–	0.08*
Total MVPA (min/week)	–	−0.11***	–	–	–	–	–	–
WC (cm)	0.11**	0.35***	−0.16***	0.18***	0.09*	–	0.17***	0.21***
Walking for travel (min)	–	–	–	–	−0.08*	–	–	–

All models initially included age, socio-economic status, subtotal fat mass, lean mass, waist circumference (WC), sitting time, total moderate–vigorous physical activity (MVPA), work MVPA, leisure MVPA, and walking for travel. Only those independent variables significant in the bivariate analysis were included in the final models shown in this table.

–, Not included in the model; HDL, high-density lipoprotein cholesterol; LDL, low-density lipoprotein cholesterol; SBP, systolic blood pressure; DBP, diastolic blood pressure.

*p < 0.05; **p < 0.005; ***p < 0.0005.

Source of data: Gradidge et al. (2014) [10].

## Results

### Patterns and correlates of self-reported physical activity

Most of the women in the study were physically active (67.0%). Overall sitting time was 180 min per day, but did not differ between the activity groups [10]. Walking for travel contributed the most to total MVPA, and was inversely associated with household SES score. Women with the highest values for walking had lower scores for SES compared with participants in the lower groups for walking [10].

Total accumulated physical activity from the various domains was higher in participants without TVs; however, leisure-time physical activity was lower in those women without cars (Figure 2) [10]. Women who owned household items that promoted sedentary behaviour reported less walking than those who did not. Leisure-time physical activity was significantly higher in those women who had cars. Sitting time was high in all groups of sedentary behaviour-promoting assets. Body composition and metabolic outcomes were similar between physically active and inactive women.

**Figure 3.**
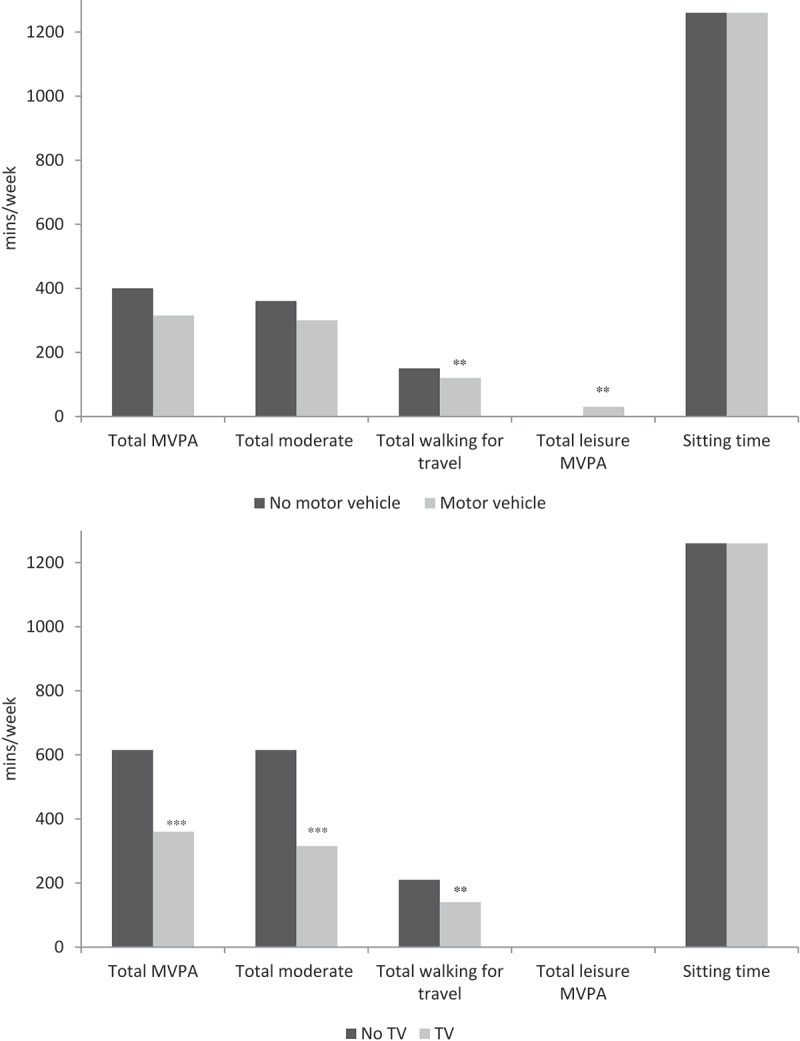
Body composition across body-size dissatisfaction groups. Source of data: Gradidge et al. (2015) [11]; ***p < 0.0005 vs women who desired to increase their body size.

### The role of behavioural and body-size perception factors in determining changes in anthropometry

The average baseline age of the participants was 41.1 ± 5.35 years. Only 3% of the participants reported smoking, and most women (83.3%) did not regularly consume alcohol. The proportion of women who did not complete high school was 50.5%, and most consumed a diet high in fatty foods more than once per week [11]. During the study period, the prevalence of obesity increased from 51% to 66%, while the prevalence of central obesity (waist circumference ≥ 80 cm) increased from 71% to 90%. All baseline measures of body composition increased significantly over the 10 year period (*p* < 0.001) [11].

Being physically active at work was associated with lean muscle mass, and age was associated with adiposity (Table 1). High-intensity (vigorous) activity was associated with smaller body composition increments, and household SES was associated with adiposity and lean mass [10]. Sitting time was associated with cardiometabolic outcomes, while walking for travel was negatively associated with total cholesterol (Table 2). Total MVPA was inversely associated with fasting insulin.

Body-composition outcomes were higher in women who had a desire to be thinner compared with those who were happy or who wanted to increase their weight (Figure 3). Most of the participants had negative scores for body-size discrepancy (74% of the participants), indicating underestimation of actual body size. The participants who had a true perception of body size and those who overestimated had lower body-composition values than those who underestimated their body size. Negative body-size discrepancy was inversely associated with peripheral fat (Table 1) [11].

Consumption of high-fat foods was not associated with the outcome measures, while cigarette smoking was associated with smaller increases in lean tissue over time. High daily intake of alcohol was associated with lower adiposity in the central region [11].

### Metabolic and anthropometric risk factors associated with MetS

The MetS increased from 40% at baseline to 49.6% in the follow-up. Table 3 shows that FFSTM in the trunk increases the risk of MetS, while subcutaneous fat in the abdominal region is associated with a lower risk of MetS. HOMA, age, and cigarette smoking are associated with an increased risk, while adiponectin is associated with a lower risk of MetS, and is the least affected by adjustment for the other risk factors (HOMA, age, cigarette smoking, and subcutaneous fat).

**Table 3.** Multivariable linear regression models for metabolic syndrome (MetS) and associated metabolic outcomes.

Covariate	Odds ratio (95% confidence interval) for outcomes				
	MetS^a^	Triglycerides^a^	HDL^a^	Glucose^a^	Elevated BP^a^
Age	1.34 (1.04, 1.16)***	1.07 (1.02, 1.12)**	–	1.09 (1.03, 1.15)**	1.05 (1.001, 1.11)*
Adiponectin (µg/ml)	0.84 (0.77, 0.92)***	0.92 (0.86, 0.98)***	0.93 (0.90, 0.97)***	0.93 (0.87, 1.001)*	–
Cigarette smoking	3.07 (1.28, 7.33)*	2.53 (1.21, 5.30) *	–	–	–
FFSTM (trunk) (kg)	1.34 (1.10, 1.61)**	–	1.14 (1.04, 1.24) *	–	–
HOMA	1.31 (1.16, 1.48)***	1.11 (1.001, 1.24)*	–	1.73 (1.49, 2.00)***	–
Leg fat (kg)	–	0.85 (0.79, 0.92)***	–	–	–
Subcutaneous abdominal fat (kg)	0.56 (0.39, 0.79)**	–	–	0.59 (0.42, 0.83) **	–

The metabolic syndrome (MetS) was defined as three or more out of four components, without waist circumference [12]. The following scientifically plausible variables were included in the initial bivariate logistic regression analysis: homeostasis model assessment (HOMA), adiponectin, trunk fat-free soft-tissue mass (FFSTM), subcutaneous abdominal fat thickness, waist and hip circumference, total body fat mass, total body FFSTM, subcutaneous and visceral adipose fat thickness, menopausal status, receiving anti-retroviral medication, and smoking. The models shown here display the independent variables that remained after backward, stepwise removal of non-significant (*p* > 0.05) variables.

^a^Cut-offs defined by the harmonised method [29].

– Not included in the model; BP, blood pressure; HDL, high-density lipoprotein cholesterol.

*p < 0.05; **p < 0.005; ***p < 0.0005.

Source of data: Gradidge et al. (2016) [12].

## Discussion

This study showed that obesity and MetS were high and associated with several key determinants, with the most influential metabolic factor in the pathogenesis of MetS being adiponectin. Behavioural and body-size perception factors are shown to be predictors of change in body composition. This discussion will focus on these key findings and propose some recommendations to address the increasing prevalence of these diseases in black South African women.

Adiponectin is an adipokine which decreases when fat accumulates [20]. In this paper, adiponectin was observed as an independent protective factor against MetS and its associated components, owing to the inverse relationship of adiponectin with triglycerides and fasting glucose, and the positive association of adiponectin with HDL cholesterol. Our findings therefore support the hypothesis that reduced concentrations of adiponectin have a role in the risk of cardiometabolic diseases [33]. Furthermore, our data also demonstrate the protective characteristics of adiponectin against dyslipidaemia and type 2 diabetes. This study also found an association of decreased abdominal subcutaneous fat with a higher risk of MetS, which has not been observed in previous studies. This intriguing relationship can be explained by the removal of waist circumference from the diagnostic criteria for MetS, which uncovered the novel relationship between subcutaneous abdominal fat and MetS. Frayn also hypothesised that this fat depot can potentially act as a protective mechanism against insulin resistance by functioning as a reservoir for initial lipid deposition, therefore delaying central adiposity [34]. However, the protective nature of subcutaneous fat against metabolic diseases needs further investigation in other African populations before Frayn’s theory can be confirmed.

Our data show that there is a coexistence of high physical activity and high sedentary behaviour in the study population, indicated by the high sitting time amid high physical activity. As a result, obesity remained high at follow-up, despite most women being classified as ‘sufficiently active’, and high sitting time was associated with several cardiometabolic diseases in this study. We also did not observe any differences in body composition and metabolic diseases between activity groups in this study, but our data on physical activity are comparable to women in other African countries [14]. Moreover, our data show that higher intensity physical activity protects against fat accumulation and the risk of cardiometabolic disorders.

Lower adiposity was related to body-size perception in this study population. Our data show that the traditional processes surrounding body-size perception in African women seem to be changing. Thus, the results highlight the existence of one obese group of women who were content with their body size, while another group of obese women had a desire to be thinner.

Smoking is uncommon among black South African or African women [2,35], and our data confirm this. We demonstrated that smoking is associated with lower lean mass. Alcohol use is also uncommon among African women [2], and our data demonstrate that the frequency of alcohol correlates with lower adiposity in the central region; however, this finding must be interpreted with caution.

The consumption of high-fat foods did not have an influence on body-composition outcomes in this study population. Our results are similar to a study of Americans, which showed that the consumption of full-cream dairy products is not associated with weight gain [36]. Further study of the comprehensive dietary pattern of our study populations is, however, needed before causality can be inferred.

### Limitations

A limitation was that dietary patterns of the study population were not included in the analyses. Therefore, we are potentially missing another important piece of the puzzle around modifiable lifestyle factors. However, these data have been collected and will form part of subsequent analyses.

### Strengths

This paper was able to draw on 10 years of secondary and prospective data from the Bt20 longitudinal cohort study [21]. The study population was large enough to be representative of black South African women living in Soweto, Johannesburg, a peri-urban setting undergoing rapid urbanisation. Furthermore, an environment of openness for sharing information pertinent to the completion of the questionnaires was created with the exclusive assistance of female, African, multilingual research assistants.

## Conclusions

Given the contemporary global focus on non-communicable diseases, targeted obesity interventions can incorporate the findings of this paper to suit a holistic contextual approach to curb the increasing presence of obesity and related metabolic diseases in black South African women. The high sitting times of obese African women who are happy with larger body size can expressly be targeted as a potential solution. Many solutions exist for targeting sitting time; however, accumulated time spent in unstructured movement or standing breaks seem the most feasible adoptable solutions in the African context [37]. Our data show that the traditional acceptance of large body size is not homogeneous in this study group, indicating that plans to address weight gain may be welcomed. There is also evidence showing that exposure to fast-food adverts on TV is associated with overweight and obesity [38], suggesting that obesity risk can be addressed by limiting screen time. The finding in this study of a strong effect of adiponectin against MetS and its various metabolic components suggests an important new metabolic factor in the aetiology of the syndrome in African women.

## Paper context

Urbanised black South African women have a high prevalence of obesity and related cardiovascular disease risk factors. In a cohort of black African women from Soweto, there is evidence that high physical activity coexists with high sitting time, with the former negative risk factor being associated with greater lean mass, and the latter positive risk factor with high adiposity and disease risk. Adiponectin, an adipose secretory hormone which falls with increasing fat mass, was shown to have a significant protective role against metabolic syndrome that is independent of other risk factors.
